# Colorectal Cancer Risk in Korean Patients with Inflammatory Bowel Disease: A Nationwide Big Data Study of Subtype and Socioeconomic Disparities

**DOI:** 10.3390/jcm14155503

**Published:** 2025-08-05

**Authors:** Kyeong Min Han, Ho Suk Kang, Joo-Hee Kim, Hyo Geun Choi, Dae Myoung Yoo, Nan Young Kim, Ha Young Park, Mi Jung Kwon

**Affiliations:** 1 Hallym Data Science Laboratory, Hallym University College of Medicine, Anyang 14068, Republic of Korea; km.han@hallym.ac.kr (K.M.H.); ydm@hallym.ac.kr (D.M.Y.); 2 Division of Gastroenterology, Department of Internal Medicine, Hallym University Sacred Heart Hospital, Hallym University College of Medicine, Anyang 14068, Republic of Korea; hskang76@hallym.or.kr; 3 Division of Pulmonary, Allergy, and Critical Care Medicine, Department of Internal Medicine, Hallym University Sacred Heart Hospital, Hallym University College of Medicine, Anyang 14068, Republic of Korea; luxjhee@hallym.or.kr; 4 Suseo Seoul E.N.T. Clinic, 10, Bamgogae-ro 1-gil, Gangnam-gu, Seoul 06349, Republic of Korea; mdanalytics@naver.com; 5 Hallym Institute of Translational Genomics and Bioinformatics, Hallym University Medical Center, Anyang 14068, Republic of Korea; honeyny@hallym.or.kr; 6 Department of Pathology, Busan Paik Hospital, Inje University College of Medicine, Busan 47392, Republic of Korea; hy08.park@gmail.com; 7 Department of Pathology, Hallym University Sacred Heart Hospital, Hallym University College of Medicine, Anyang 14068, Republic of Korea

**Keywords:** colorectal cancer, inflammatory bowel disease, ulcerative colitis, Crohn’s disease, nationwide population-based study, big data, Korea

## Abstract

**Background/Objectives**: The two major subtypes of inflammatory bowel disease (IBD)—Crohn’s disease (CD) and ulcerative colitis (UC)—are known to increase the likelihood of developing colorectal cancer (CRC). While this relationship has been well studied in Western populations, evidence from East Asia remains limited and inconsistent. Using nationwide cohort data, this study explored the potential connection between IBD and CRC in a large Korean population. **Methods**: We conducted a retrospective cohort study using data from the Korean National Health Insurance Service–National Sample Cohort from 2005 to 2019. A total of 9920 CRC patients were matched 1:4 with 39,680 controls using propensity scores based on age, sex, income, and region. Overlap weighting and multivariable logistic regression were used to evaluate the association between IBD and CRC. Subgroup analyses were conducted to assess effect modification by demographic and clinical factors. **Results**: IBD markedly increased the likelihood of developing CRC (adjusted odds ratio (aOR) = 1.38; 95% confidence interval (CI): 1.20–1.58; *p* < 0.001), with the association primarily driven by UC (aOR = 1.52; 95% CI: 1.27–1.83). CD appeared unrelated to heightened CRC risk overall, though a significant association was observed among low-income CD patients (aOR = 1.58; 95% CI: 1.15–2.16). The UC–CRC association persisted across all subgroups, including patients without comorbidities. **Conclusions**: Our findings support an independent association between IBD—particularly UC—and increased CRC risk in Korea. These results underscore the need for personalized CRC surveillance strategies that account for disease subtype, comorbidity burden, and socioeconomic status, especially in vulnerable subpopulations.

## 1. Introduction

Colorectal cancer (CRC) is the third most commonly diagnosed cancer and the second leading cause of cancer-related mortality worldwide, accounting for approximately 10% of all cancer cases and deaths [1]. South Korea ranks among the highest in CRC incidence globally, with 44.5 cases per 100,000 individuals annually, making CRC the third most prevalent cancer in the country [2]. This rise has paralleled rapid socioeconomic development, urbanization, and lifestyle changes such as dietary Westernization, reduced physical activity, and increasing obesity [3,4,5].

Inflammatory bowel disease (IBD), which includes ulcerative colitis (UC) and Crohn’s disease (CD), is a condition of persistent and recurring inflammation affecting the digestive tract that is increasing in prevalence across the Asia–Pacific region, including Korea [6,7,8,9,10,11]. UC typically involves continuous mucosal inflammation beginning in the rectum and extending proximally, while CD may affect any part of the gastrointestinal tract with a transmural and segmental pattern [6,7]. Historically considered a Western disease, IBD incidence in Korea has grown significantly over the past three decades, with UC and CD incidence rising from 0.29 to 5.82 and 0.06 to 2.44 per 100,000, respectively [8,9,12].

Colitis-associated CRC is one of the most serious complications of long-standing IBD, particularly UC [13]. Chronic mucosal inflammation in IBD is believed to contribute to CRC through molecular alterations including TP53 and APC mutations, microsatellite instability, and dysplasia–carcinoma progression [14]. While earlier studies in Western populations reported cumulative CRC risks of 2% at 10 years, 8% at 20 years, and up to 18% at 30 years after IBD diagnosis [15], more recent meta-analyses suggest lower but still elevated risks in patients with extensive or long-duration disease [16]. In contrast, Asian studies have reported a lower prevalence of IBD-associated CRC (0.1–1.8%) [9,13,17], although findings remain inconsistent.

Recent Korean and East Asian studies have yielded mixed results. A tertiary hospital-based study in Korea reported rising UC-related colorectal neoplasia incidence following national reimbursement for biologics [18], while another Korean cohort found increased CRC risk in both UC and CD [19]. In contrast, a Hong Kong multicenter study identified increased cancer risk in CD (especially anorectal and hematologic malignancies) but not in CRC [20], and a Taiwanese study found elevated CRC risk in CD but not UC [21]. These differences may reflect variations in study design, sample sizes, demographic and clinical characteristics, surveillance practices, and healthcare access. Importantly, most of these studies are limited by hospital-based cohorts, referral bias, and insufficient adjustment for confounders such as socioeconomic status.

While IBD is a recognized CRC risk factor [14], few studies have explored how CRC risk differs by IBD subtype and across socioeconomic strata in Asian populations. Given Korea’s universal health coverage and extensive national claims databases, the Korean setting offers a unique opportunity for population-based analysis. Socioeconomic factors such as income and healthcare access may influence CRC risk through differences in diagnosis, surveillance uptake, and treatment but have been underexplored in IBD-related CRC research in Asia [22,23,24].

In this study, we aimed to evaluate the differential risk of CRC in patients with UC and CD, with a specific focus on demographic and socioeconomic disparities. Improved understanding of these factors may support accurate risk stratification and guide more tailored surveillance strategies for high-risk IBD subgroups in clinical and public health settings.

## 2. Materials and Methods

### 2.1. Ethics

This research received clearance from the Institutional Review Board of Hallym University (Approval No: 2022-10-008) and was conducted in accordance with the principles of the Declaration of Helsinki. Owing to the use of pre-existing administrative data, the board exempted the need for obtaining written participant consent. All procedures adhered to the ethical standards and regulatory framework established by the Hallym University Institutional Review Board, complied with national ethical guidelines for observational studies, and were reported in accordance with the STROBE (Strengthening the Reporting of Observational Studies in Epidemiology) statement.

### 2.2. Research Design and Participant Enrollment

This study utilized data from the Korean National Health Insurance Service-National Sample Cohort (NHIS-NSC) which includes participants from 2002 to 2019 by the NHIS in the Republic of Korea [25]. The NHIS-NSC includes a simple random sample representing 2.2% of the eligible Korean population in 2002, and participants were followed until 2019 unless they were disqualified due to death or emigration. The dataset was segmented into 1476 subgroups according to demographic factors, including age (classified into 18 segments: newborns under 1 year, toddlers aged 1–4, successive 5-year brackets from 5 to 79 years, and those aged 80 and older), biological sex (male or female), and economic standing (divided into 41 tiers: 20 quantiles for salaried workers, 20 for self-employed individuals, and the lowest level for public assistance recipients).

From a total of 1,137,861 participants and 219,673,817 medical claim codes recorded between 2005 and 2019, we identified 9920 patients with CRC. The control group comprised individuals who were not diagnosed with CRC during the same period (*n* = 1,127,941), after which 3472 individuals who had any record of CRC diagnosis were excluded.

**Figure 1:**
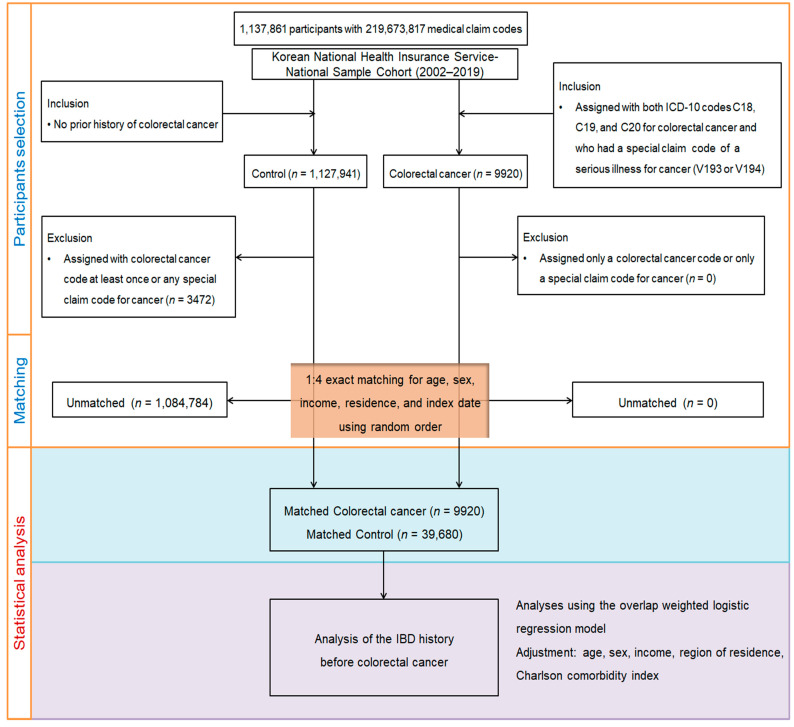
Flowchart of participant selection and study design. This study analyzed data from the Korean National Health Insurance Service–National Sample Cohort (2002–2019), which includes 1,137,861 individuals and over 219 million medical claims. Colorectal cancer (CRC) cases were identified using ICD-10 codes (C18, C19, C20) along with a special claims code for serious cancer (V193 or V194). Controls were selected from individuals with no history of CRC or cancer-related claims. After exclusions, 1:4 exact propensity score matching was performed based on age, sex, income, region, and index date. The final sample included 9920 CRC cases and 39,680 matched controls. Then, inflammatory bowel disease (IBD) history was assessed prior to CRC diagnosis.

To improve the accuracy and reduce potential bias, each CRC patient was matched to four controls via 1:4 propensity score matching on the basis of age, sex, income level, and region of residence. Comparison individuals were randomly selected in order to minimize selection bias. For each CRC patient, the date of initial treatment was defined as the index date. The same index date was assigned to the matched control participants to ensure consistency. In the course of the pairing procedure, a total of 1,084,784 comparison subjects were omitted. Consequently, 39,680 comparator individuals were retained for inclusion in the conclusive evaluation (Figure 1).

### 2.3. Definition of Colorectal Cancer (Outcome)

To ensure diagnostic accuracy and exclude false-positive cases, CRC was identified via specific ICD-10 codes: C18 (malignant neoplasm of the colon), C19 (rectosigmoid junction), C20 (rectum). Among individuals with these diagnostic codes, only those with accompanying special claims codes for cancer (V193 or V194) were classified as CRC cases. These special claim codes indicate confirmed cancer diagnoses and eligibility for reduced health care costs due to the severity of the disease.

### 2.4. Definition of Inflammatory Bowel Disease (Exposure)

IBD was defined as a diagnosis of either Crohn’s disease (CD; ICD-10: K50) or ulcerative colitis (UC; ICD-10: K51). In this study, participants diagnosed with either CD or UC were considered to have IBD.

### 2.5. Covariates

Subjects were stratified into 18 distinct age brackets in 5-year increments. Economic standing was segmented into five tiers, from category 1 (lowest quintile) to category 5 (highest quintile). Geographical regions were divided into 16 local government units and further classified as metropolitan or non-metropolitan. Metropolitan areas encompassed the seven most populous Korean cities (Seoul, Incheon, Daejeon, Daegu, Gwangju, Ulsan, and Busan), each with more than one million inhabitants. Non-metropolitan areas included less densely populated provinces such as Chungcheongbuk-do, Chungcheongnam-do, Gyeonggi-do, Gangwon-do, Gyeongsangbuk-do, Gyeongsangnam-do, Jeollabuk-do, Jeollanam-do, and Jeju. The Charlson Comorbidity Index (CCI) was applied to quantify the overall burden of chronic conditions, yielding a composite score from 0 to 29 based on 17 diagnostic categories [26]. To isolate the effects of other comorbidities on colorectal cancer development, cancer was omitted from the CCI score in this study.

### 2.6. Statistical Analyses

Propensity score overlap weighting was employed to balance baseline characteristics between the CRC and control groups, thereby improving the precision of the analysis. Propensity scores were estimated via multivariable logistic regression including all covariates. We chose overlap weighting method because it mimics randomized trials and improves efficiency in observational studies. For the calculation of overlap weights, a weight of (1 − propensity score) was assigned to the CRC group and a weight of propensity score was assigned to the control group [27]. Standardized mean differences were used to assess the balance of covariates between the two groups [28].

To estimate the odds ratios (ORs) and corresponding 95% confidence intervals (CIs) regarding the link between prior IBD diagnosis and subsequent CRC onset, multivariable logistic regression models incorporating overlap weighting were applied. Both unadjusted (baseline) and covariate-adjusted models were analyzed, with adjustments accounting for demographic and clinical variables including age, gender, socioeconomic status, residential area, and CCI. Stratified analyses were conducted across all prespecified covariate categories. All inferential procedures were two-tailed, with statistical significance defined as a *p*-value below 0.05. Data processing and analysis were carried out using SAS software, version 9.4 (SAS Institute, Cary, NC, USA).

## 3. Results

### 3.1. Baseline Characteristics

Table 1 outlines the initial profiles of participants prior to and following the application of propensity score overlap weighting. The analysis comprised 9920 individuals with CRC and 39,680 corresponding controls matched accordingly. The study groups were balanced across demographic and socioeconomic factors including age, sex, income level, and residential region (each standardized difference = 0.00). Following the implementation of overlap weighting to balance potential confounding factors, the absolute standardized differences for all included variables were reduced to below 0.2, indicating adequate covariate equilibrium between the colorectal cancer cohort and the comparator group.

### 3.2. Relationship Between Inflammatory Bowel Diseases and Colorectal Cancer

We examined the potential association between IBD and the incidence of CRC in comparison to a matched control group (Table 2). Overall, a significantly greater proportion of CRC patients had IBD compared to controls (1.90% vs. 1.37%). In the overlap-weighted multivariable logistic regression analysis, IBD was significantly associated with increased odds of developing CRC (adjusted OR = 1.38; 95% CI = 1.20–1.58; *p* < 0.001).

**Figure 2:**
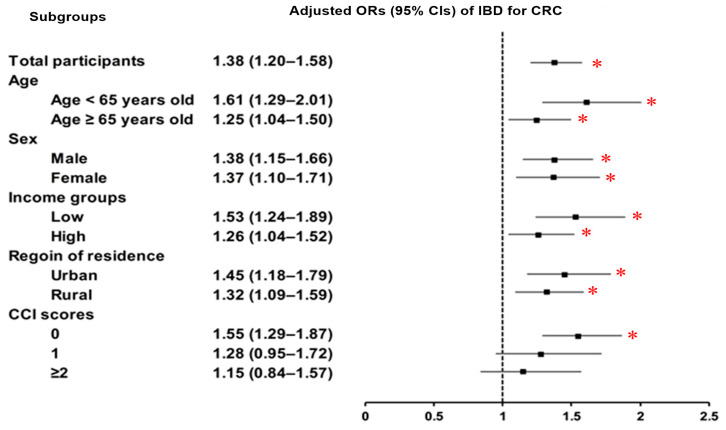
Subgroup analysis of IBD and CRC risk stratified by age, sex, income, residence, and CCI. Forest plots demonstrate a consistent association between IBD and CRC across all subgroups, including patients without comorbidities (CCI = 0). Abbreviations: IBD, inflammatory bowel disease; CRC, colorectal cancer; OR, odds ratios; 95% CI, 95% confidence interval; CCI, Charlson Comorbidity Index. * Significance at *p* < 0.05.

To explore this association further, subgroup analyses were conducted based on demographic and clinical characteristics. These analyses consistently demonstrated that the association between IBD and CRC remained significant across subgroups defined by sex, age, income level, residential region, and in patients with no comorbidities (CCI score = 0), reinforcing the robustness of the observed relationship (Figure 2 and Supplementary Table S1).

### 3.3. Relationship Between Crohn’s Disease and Colorectal Cancer

The proportions of CRC among patients with CD and the control group were 0.75% and 0.63%, respectively. However, both the crude and adjusted ORs indicated no statistically significant difference in CRC risk between the two groups (crude OR = 1.19; 95% CI = 0.92–1.54; *p* = 0.190; adjusted OR = 1.18; 95% CI = 0.95–1.45; *p* = 0.139).

**Figure 3:**
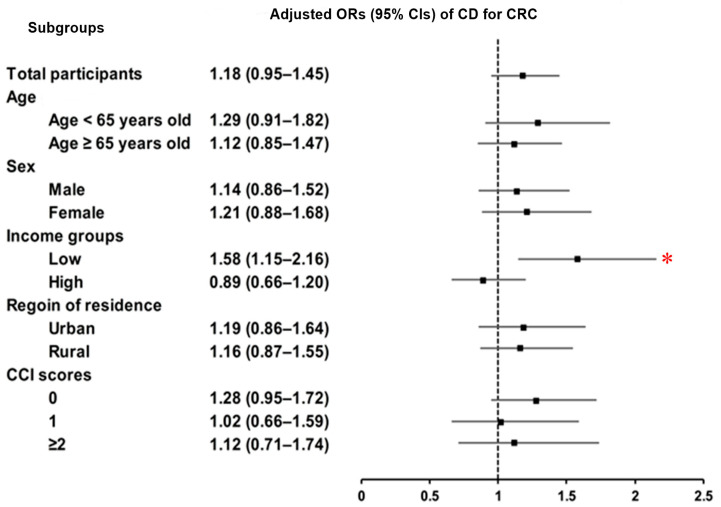
Subgroup analyses of CD and CRC by age, sex, income, region, and CCI score. The forest plots showed no significant association between CD and CRC in most subgroups, except among low-income individuals, where CD was significantly associated with a higher CRC risk. Abbreviations: CD, Crohn’s Disease; CRC, colorectal cancer; OR, odds ratios; 95% CI, 95% confidence interval; CCI, Charlson Comorbidity Index. * Significance at *p* < 0.05.

Likewise, stratified evaluations by various factors showed no meaningful link between CD and CRC risk, except in the group with lower socioeconomic status. In this group, CD was significantly associated with a higher likelihood of CRC (adjusted OR = 1.58; 95% CI = 1.15–2.16; *p* = 0.004), even after adjustment for confounders (Figure 3 and Supplementary Table S2). However, the interaction between income group and CD status was not statistically significant (*p* for interaction = 0.27).

### 3.4. Relationship Between Ulcerative Colitis and Colorectal Cancer

**Figure 4:**
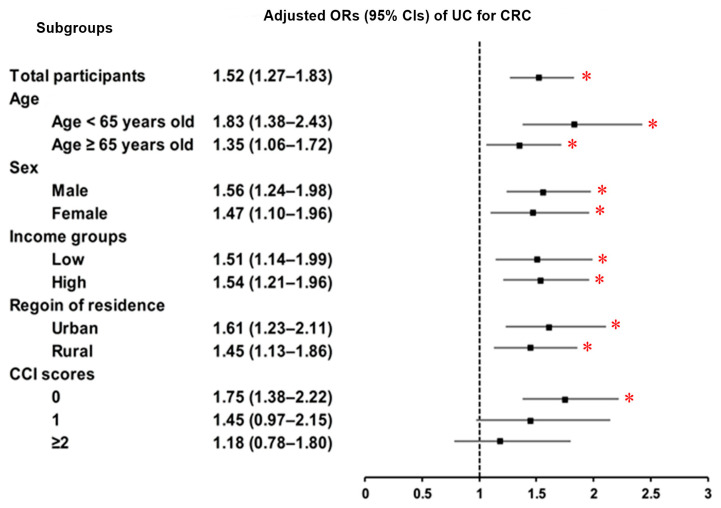
Subgroup analyses of UC and CRC by age, sex, income, region, and CCI score. Forest plots showed a consistently significant association between UC and increased CRC risk across most subgroups, including all ages, sexes, income levels, regions, and among patients without comorbidities (CCI = 0). Abbreviations: UC, ulcerative colitis; CRC, colorectal cancer; OR, odds ratios; 95% CI, 95% confidence interval; CCI, Charlson Comorbidity Index. * Significance at *p* < 0.05.

We also examined the association between UC and the incidence of CRC compared to controls. UC was identified in 1.18% of CRC patients and 0.78% of controls, demonstrating a strong and statistically significant association with increased odds of CRC (adjusted OR = 1.52; 95% CI = 1.27–1.83; *p* < 0.001). This association remained consistent across most subgroups, regardless of sex, age, income level, region of residence, or comorbidity status (CCI score = 0), as illustrated in the forest plot (Figure 4 and Supplementary Table S3).

## 4. Discussion

In this large, nationwide Korean cohort study, we observed a statistically significant association between IBD and CRC, with individuals diagnosed with IBD demonstrating a 38% higher likelihood of developing CRC compared to matched controls. This increased risk remained consistent across subgroups stratified by age, sex, income level, region of residence, and comorbidity burden, suggesting that IBD may serve as an independent risk factor for CRC in the Korean population. Notably, this risk was predominantly attributable to UC, which was associated with a 52% increased risk of CRC (adjusted OR = 1.52; 95% CI = 1.27–1.83), while CD did not show a significant overall association. These findings have important clinical implications, suggesting that CRC surveillance strategies in patients with IBD should be tailored based on disease subtype and patient-specific factors. In particular, comorbidity burden in UC and socioeconomic status in CD may help identify subpopulations at greater risk. Overall, our results provide epidemiological support for the link between IBD and CRC and highlight the need for targeted preventive approaches in high-risk groups.

Our findings are partially consistent with prior data from Asia, while offering novel insights derived from a robust, population-based design. Reported prevalence rates of IBD-related CRC in Asian countries—including Korea, Japan, Hong Kong, and India—range from 0.1% to 1.8% [9,10,17,20], closely aligning with the 1.90% prevalence observed among CRC patients in our cohort. Similarly, a population-based study from Japan identified 169 IBD-related CRC cases, reporting no sex-specific differences and a mean age at diagnosis of 56.8 years [11]. Although IBD-associated CRC has traditionally been considered more common in younger individuals, emerging evidence, including our results, suggests that older patients also face increased risk [29]. Additionally, CRC risk appears to be higher among IBD patients with comorbid conditions [29]. Interestingly, IBD-associated CRC patients with CD or UC have been reported to often reside in higher-income areas, have private insurance, and exhibit fewer comorbidities than non-IBD CRC patients [30]. These patterns, consistent with our findings, underscore the importance of individualized risk stratification for optimizing CRC surveillance in IBD populations.

We found that not all individuals with IBD shared the same level of CRC risk. Stratified analysis revealed that patients with UC had a significantly increased likelihood of developing CRC (adjusted OR = 1.52; 95% CI = 1.27–1.83; *p* < 0.001). The CRC prevalence in UC patients within our cohort was 1.18%, which aligns with previously reported rates in the Asia–Pacific region (0.1–1.8%) [17], but remains lower than those reported in Western populations, where UC-associated CRC prevalence ranges from 0.3% to 1.8%, and the relative risk is estimated to be between 0.9 and 8.3 times that of the general population [16,31,32,33]. In contrast, a Taiwanese population-based study reported an elevated CRC risk in CD but not in UC patients [21], while a Hong Kong multicenter study found increased cancer risk in CD—particularly anorectal and hematologic malignancies—but did not demonstrate a consistent association with CRC [20]. These discrepancies may reflect differences in demographic and clinical characteristics, healthcare systems, environmental exposures, surveillance practices, and genetic predispositions. For example, the small and unbalanced CD sample size (*n* = 685) compared to UC (*n* = 2663) in the Taiwanese study could have introduced imbalance and limited statistical power [21]. The Hong Kong study also assessed a broad spectrum of cancers, which may have diluted CRC-specific associations due to limited case numbers [20]. Regional variation in IBD incidence may further explain these differences. Taiwan reported a lower IBD incidence (1.38 per 100,000 persons/year from 2010 to 2013) compared to Japan or Korea [8,9,10,21], potentially contributing to lower CRC detection rates. Our findings suggest that CRC risk in UC patients may vary regionally and underscore the need for tailored surveillance strategies based on local epidemiologic data rather than universal application of Western guidelines. Notably, CRC surveillance in IBD patients is not clearly addressed in current Asia–Pacific consensus guidelines [9]. Our results support adapting Western recommendations—such as initiating CRC surveillance 8 to 10 years after UC diagnosis—for East Asian populations [12,14,34,35]. High-risk groups, particularly those with UC, should be prioritized for more intensive CRC screening.

Notably, the association between UC and increased CRC risk remained consistent across most subgroups, regardless of age, sex, income level, or region of residence. Interestingly, we also observed a 75% increased likelihood of CRC development (adjusted OR = 1.75; 95% CI = 1.38–2.22; *p* < 0.001) among UC patients without comorbidities, suggesting that disease-related factor itself may independently drive carcinogenesis. While previous studies have reported increased prevalence of comorbidities—such as chronic ischemic heart disease, diabetes mellitus, and obesity (included in CCI score analyses), as well as primary sclerosing cholangitis (not included in the CCI)—among UC patients with CRC [29], our findings highlight that UC remains a significant clinical predictor of CRC even without these risk factors. This underscores the need for vigilant CRC surveillance in UC patients, including those with no measurable comorbidity burden (CCI = 0). However, the potential for detection bias should be considered. IBD patients typically undergo more frequent endoscopic evaluations than the general population, which could increase the likelihood of CRC detection and possibly lead to overestimation of relative risk [36]. Conversely, limited access to timely diagnosis and surveillance colonoscopy may elevate CRC risk in vulnerable subgroups, particularly those facing healthcare disparities [12]. These findings underscore the importance of ensuring equitable access to CRC surveillance, even for patients without additional comorbidities, when developing screening protocols.

In this study, CD was not associated with an increased overall risk of CRC. This finding may reflect the relatively low prevalence of CD in the Asia–Pacific region, despite a gradual rise in incidence over recent decades [9,14]. For example, a 20-year retrospective study in Japan involving 294 CD patients identified only 13 cancer cases, including 6 CRCs, with cumulative incidence rates of 0.25% at 10 years and 0.58% at both 15 and 20 years [37]. Similarly, a population-based study in Hong Kong reported increased risks of certain malignancies in CD (notably anorectal and hematologic cancers) but no consistent elevation in CRC risk [20]. These findings align with our results, suggesting that CRC risk in CD may not be universally elevated. The absence of a significant association may also reflect variations in disease localization or disparities in healthcare access. As CD frequently affects the small intestine rather than the colon, its direct contribution to colonic neoplasia may be limited unless there is substantial colonic involvement.

Although CD was not associated with a significantly increased overall risk of CRC, stratified analysis revealed that CD patients in the low-income group had a significantly higher CRC risk. While the interaction between income and CD was not statistically significant, this finding highlights the potential vulnerability of socioeconomically disadvantaged patients. IBD patients with low socioeconomic status may face substantial barriers to receiving appropriate and adequate medical care, including access to timely diagnosis, regular follow-up, and colonoscopic surveillance, which may contribute to an increased risk of CRC. These individuals are more likely to experience delayed detection and poorer outcomes due to limited access to preventive services and continuity of care. Although causality cannot be inferred, our results underscore the importance of improving access to preventive care and CRC screening for underserved populations with CD.

A key strength of this study is the use of a large, nationally representative Korean cohort with long-term follow-up, which enabled a robust assessment of colorectal cancer risk in IBD patients. Additionally, propensity score matching minimized confounding and improved comparability between groups. This study has several limitations. First, its retrospective observational design limits our ability to establish a definitive causal relationship between IBD and CRC. Second, the analysis was restricted to the Korean population and relied on diagnostic codes from the National Health Insurance database, which may have introduced misclassification bias and excluded unmeasured confounding variables. These factors may limit the generalizability of our findings to other populations or healthcare systems. Third, the NHIS-NSC database lacks detailed clinical information, such as the severity and duration of IBD, CRC staging, tumor histology, family history, and genetic predispositions. As a result, we were unable to assess how these potentially important variables may have influenced CRC risk, representing an area for future research.

## 5. Conclusions

In this nationwide cohort study, we found that CRC risk among Korean patients with IBD differs by disease subtype, age, sex, and socioeconomic status. The highest risk was observed in patients with UC, particularly older males and those from lower-income groups. While our study does not explore mechanistic pathways, the identification of high-risk subgroups using real-world data may support more targeted CRC surveillance approaches. Further research incorporating clinical, genetic, and treatment-related factors will be essential to refine personalized screening strategies for IBD patients.

## Figures and Tables

**Table 1:** General Characteristics of Participants.

Characteristics	Before PS Overlap Weighting Adjustment			After PS Overlap Weighting Adjustment		
	CRC	Control	Standardized Difference	CRC	Control	Standardized Difference
Age (y), *n* (%)			0.00			0.00
0–4	1 (0.01)	4 (0.01)		1 (0.01)	1 (0.01)	
5–9	N/A	N/A		N/A	N/A	
10–14	3 (0.03)	12 (0.03)		2 (0.03)	2 (0.03)	
15–19	1 (0.01)	4 (0.01)		1 (0.01)	1 (0.01)	
20–24	8 (0.08)	32 (0.08)		6 (0.08)	6 (0.08)	
25–29	26 (0.26)	104 (0.26)		21 (0.26)	21 (0.26)	
30–34	94 (0.95)	376 (0.95)		75 (0.95)	75 (0.95)	
35–39	180 (1.81)	720 (1.81)		144 (1.81)	144 (1.81)	
40–44	359 (3.62)	1436 (3.62)		287 (3.61)	287 (3.61)	
45–49	578 (5.83)	2312 (5.83)		462 (5.82)	462 (5.82)	
50–54	968 (9.76)	3872 (9.76)		772 (9.75)	772 (9.75)	
55–59	1242 (12.52)	4968 (12.52)		992 (12.51)	992 (12.51)	
60–64	1393 (14.04)	5572 (14.04)		1112 (14.02)	1112 (14.02)	
65–69	1488 (15.00)	5952 (15.00)		1189 (15.00)	1189 (15.00)	
70–74	1471 (14.83)	5884 (14.83)		1176 (14.84)	1176 (14.84)	
75–79	1060 (10.69)	4240 (10.69)		848 (10.70)	848 (10.70)	
80–84	672 (6.77)	2688 (6.77)		539 (6.80)	539 (6.80)	
85+	376 (3.79)	1504 (3.79)		301 (3.80)	301 (3.80)	
Sex, *n* (%)			0.00			0.00
Male	5933 (59.81)	23,732 (59.81)		4739 (59.79)	4739 (59.79)	
Female	3987 (40.19)	15,948 (40.19)		3187 (40.21)	3187 (40.21)	
Income, *n* (%)			0.00			0.00
1 (lowest)	1990 (20.06)	7960 (20.06)		1590 (20.06)	1590 (20.06)	
2	1253 (12.63)	5012 (12.63)		1000 (12.62)	1000 (12.62)	
3	1562 (15.75)	6248 (15.75)		1247 (15.74)	1247 (15.74)	
4	2059 (20.76)	8236 (20.76)		1646 (20.77)	1647 (20.77)	
5 (highest)	3056 (30.81)	12,224 (30.81)		2442 (30.81)	2442 (30.81)	
Region of residence, *n* (%)			0.00			0.00
Urban	4447 (44.83)	17,788 (44.83)		3553 (44.83)	3553 (44.83)	
Rural	5473 (55.17)	21,892 (55.17)		4373 (55.17)	4373 (55.17)	
CCI score, mean (SD)	0.80 (1.18)	0.69 (1.18)	0.09	0.77 (1.03)	0.77 (0.57)	0.00
IBD, *n* (%)	188 (1.90)	544 (1.37)	0.04	150 (1.89)	110 (1.38)	0.04
CD, *n* (%)	74 (0.75)	249 (0.63)	0.01	59 (0.74)	50 (0.63)	0.04
UC, *n* (%)	117 (1.18)	306 (0.77)	0.04	94 (1.18)	62 (0.78)	0.01

Abbreviations: PS, propensity score; CRC, colorectal cancer; N/A, not applicable; CCI, Charlson Comorbidity Index; SD, standard deviation; IBD, inflammatory bowel disease; CD, Crohn’s disease; UC, ulcerative colitis.

**Table 2:** Crude and overlap propensity score weighted odds ratios of inflammatory bowel disease, Crohn’s disease, and ulcerative colitis for colorectal cancer.

Characteristics	N of Colorectal Cancer	N of Control	OR for Colorectal Cancer (95% CI)			
	(Exposure/Total, %)	(Exposure/Total, %)	Crude	*p*	Overlap Weighted Model ^†^	*p*
Inflammatory Bowel Disease						
IBD	188/9920 (1.90)	544/39,680 (1.37)	1.39 (1.18–1.64)	<0.001 *	1.38 (1.20–1.58)	<0.001 *
Non-IBD	9732/9920 (98.1)	39,136/39,680 (98.63)	1		1	
Crohn’s Disease						
CD	74/9920 (0.75)	249/39,680 (0.63)	1.19 (0.92–1.54)	0.190	1.18 (0.95–1.45)	0.139
Non-CD	9846/9920 (99.25)	39,431/39,680 (99.37)	1		1	
Ulcerative Colitis						
UC	117/9920 (1.18)	306/39,680 (0.77)	1.54 (1.24–1.90)	<0.001 *	1.52 (1.27–1.83)	<0.001 *
Non-UC	9803/9920 (98.82)	39,374/39,680 (99.23)	1		1	

Abbreviations: OR, odds ratios; CIs, confidence intervals. ^†^ Adjusted for age, sex, income, region of residence, and CCI scores. * Significance at *p* < 0.001.

## Data Availability

Restrictions apply to the availability of these data. The data were obtained from the Korean National Health Insurance Sharing Service (NHISS) and are available at https://nhiss.nhis.or.kr, accessed on 25 June 2025.

## Supplementary Materials

The following supporting information can be downloaded at https://www.mdpi.com/article/10.3390/jcm14155503/s1: Table S1: Crude and overlap propensity score weighted odds ratios of IBD for colorectal cancer; Table S2: Crude and overlap propensity score weighted odds ratios of CD for colorectal cancer; Table S3: Crude and overlap propensity score weighted odds ratios of UC for colorectal cancer.
