# Supplementary material for: Colorectal Cancer Risk in Korean Patients with Inflammatory Bowel Disease: A Nationwide Big Data Study of Subtype and Socioeconomic Disparities
Source: J Clin Med. 2025 Aug 5;14(15):5503. doi: 10.3390/jcm14155503 (PMC12347564; doi:10.3390/jcm14155503)
Supplement: Supplementary file 1 [file jcm-14-05503-s001.zip › jcm-3748861-supplementary.pdf]

Supplementary Table S1. Crude and overlap propensity score weighted odd ratios of IBD for colorectal cancer

Characteristics	N of Colorectal cancer (exposure/total, %)	N of Control (exposure/total, %)	Odd ratios for Colorectal cancer (95% confidence interval)			
			Crude†	*p*	Overlap weighted model †	*p*
Total participants (n = 49,600)						
IBD	188/9,920 (1.9)	544/39,680 (1.37)	1.39 (1.18-1.64)	<0.001*	1.38 (1.20-1.58)	<0.001*
Control	9,732/9,920 (98.1)	39,136/39,680 (98.63)	1		1	
Age < 65 years old (n = 24,265)						
IBD	83/4,853 (1.71)	207/19,412 (1.07)	1.62 (1.25-2.09)	<0.001*	1.61 (1.29-2.01)	<0.001*
Control	4,770/4,853 (98.29)	19,205/19,412 (98.93)	1		1	
Age ≥ 65 years old (n = 25,335)						
IBD	105/5,067 (2.07)	337/20,268 (1.66)	1.25 (1.00-1.56)	0.047*	1.25 (1.04-1.50)	0.017*
Control	4,962/5,067 (97.93)	19,931/20,268 (98.34)	1		1	
Male (n = 29,665)						
IBD	111/5,933 (1.87)	320/23,732 (1.35)	1.40 (1.12-1.73)	0.003*	1.38 (1.15-1.66)	<0.001*
Control	5,822/5,933 (98.13)	23,412/23,732 (98.65)	1		1	
Female (n = 19,935)						
IBD	77/3,987 (1.93)	224/15,948 (1.4)	1.38 (1.06-1.80)	0.015*	1.37 (1.10-1.71)	0.005*
Control	3,910/3,987 (98.07)	15,724/15,948 (98.6)	1		1	
Low income group (n = 24,025)						
IBD	89/4,805 (1.85)	231/19,220 (1.2)	1.55 (1.21-1.99)	<0.001*	1.53 (1.24-1.89)	<0.001*
Control	4,716/4,805 (98.15)	18,989/19,220 (98.8)	1		1	
High income group (n = 25,575)						
IBD	99/5,115 (1.94)	313/20,460 (1.53)	1.27 (1.01-1.60)	0.04*	1.26 (1.04-1.52)	0.016*
Control	5,016/5,115 (98.06)	20,147/20,460 (98.47)	1		1	
Urban resident (n = 22,235)						
IBD	88/4,447 (1.98)	242/17,788 (1.36)	1.46 (1.14-1.87)	0.002*	1.45 (1.18-1.79)	<0.001*
Control	4,359/4,447 (98.02)	17,546/17,788 (98.64)	1		1	
Rural resident (n = 27,365)						
IBD	100/5,473 (1.83)	302/21,892 (1.38)	1.33 (1.06-1.67)	0.014*	1.32 (1.09-1.59)	0.004*
Control	5,373/5,473 (98.17)	21,590/21,892 (98.62)	1		1	
CCI scores = 0 (n = 30,455)						
IBD	101/5,448 (1.85)	300/25,007 (1.2)	1.56 (1.24-1.95)	<0.001*	1.55 (1.29-1.87)	<0.001*
Control	5,347/5,448 (98.15)	24,707/25,007 (98.8)	1		1	
CCI scores = 1 (n = 10,619)						
IBD	49/2,600 (1.88)	123/8,019 (1.53)	1.23 (0.88-1.72)	0.219	1.28 (0.95-1.72)	0.11
Control	2,551/2,600 (98.12)	7,896/8,019 (98.47)	1		1	
CCI scores ≥ 2 (n = 8,526)						
IBD	38/1,872 (2.03)	121/6,654 (1.82)	1.12 (0.77-1.62)	0.55	1.15 (0.84-1.57)	0.382
Control	1,834/1,872 (97.97)	6,533/6,654 (98.18)	1		1	

Abbreviations: IBD, inflammatory bowel disease; CCI, Charlson Comorbidity Index. * Significance at $p < 0.05$

† Adjusted for age, sex, income, region of residence, and CCI scores.

Supplementary Table S2. Crude and overlap propensity score weighted odd ratios of Crohn's disease for colorectal cancer

Characteristics	N of Colorectal cancer (exposure/total, %)	N of Control (exposure/total, %)	Odd ratios for Colorectal cancer (95% confidence interval)			
			Crude†	*p*	Overlap weighted model †	*p*
Total participants (n = 49,600)						
Crohn's disease	74/9,920 (0.75)	249/39,680 (0.63)	1.19 (0.92-1.54)	0.19	1.18 (0.95-1.45)	0.139
Control	9,846/9,920 (99.25)	39,431/39,680 (99.37)	1		1	
Age < 65 years old (n = 24,265)						
Crohn's disease	30/4,853 (0.62)	91/19,412 (0.47)	1.32 (0.87-2.00)	0.188	1.29 (0.91-1.82)	0.154
Control	4,823/4,853 (99.38)	19,321/19,412 (99.53)	1		1	
Age ≥ 65 years old (n = 25,335)						
Crohn's disease	44/5,067 (0.87)	158/20,268 (0.78)	1.11 (0.80-1.56)	0.525	1.12 (0.85-1.47)	0.432
Control	5,023/5,067 (99.13)	20,110/20,268 (99.22)	1		1	
Male (n = 29,665)						
Crohn's disease	41/5,933 (0.69)	141/23,732 (0.59)	1.16 (0.82-1.65)	0.393	1.14 (0.86-1.52)	0.359
Control	5,892/5,933 (99.31)	23,591/23,732 (99.41)	1		1	
Female (n = 19,935)						
Crohn's disease	33/3,987 (0.83)	108/15,948 (0.68)	1.22 (0.83-1.81)	0.311	1.21 (0.88-1.68)	0.237
Control	3,954/3,987 (99.17)	15,840/15,948 (99.32)	1		1	
Low income group (n = 24,025)						
Crohn's disease	41/4,805 (0.85)	102/19,220 (0.53)	1.61 (1.12-2.32)	0.01*	1.58 (1.15-2.16)	0.004*
Control	4,764/4,805 (99.15)	19,118/19,220 (99.47)	1		1	
High income group (n = 25,575)						
Crohn's disease	33/5,115 (0.65)	147/20,460 (0.72)	0.90 (0.61-1.31)	0.575	0.89 (0.66-1.20)	0.449
Control	5,082/5,115 (99.35)	20,313/20,460 (99.28)	1		1	
Urban resident (n = 22,235)						
Crohn's disease	33/4,447 (0.74)	110/17,788 (0.62)	1.20 (0.81-1.78)	0.357	1.19 (0.86-1.64)	0.288
Control	4,414/4,447 (99.26)	17,678/17,788 (99.38)	1		1	
Rural resident (n = 27,365)						
Crohn's disease	41/5,473 (0.75)	139/21,892 (0.63)	1.18 (0.83-1.68)	0.35	1.16 (0.87-1.55)	0.301
Control	5,432/5,473 (99.25)	21,753/21,892 (99.37)	1		1	
CCI scores = 0 (n = 30,455)						
Crohn's disease	36/5,448 (0.66)	128/25,007 (0.51)	1.29 (0.89-1.87)	0.174	1.28 (0.95-1.72)	0.101
Control	5,412/5,448 (99.34)	24,879/25,007 (99.49)	1		1	
CCI scores = 1 (n = 10,619)						
Crohn's disease	20/2,600 (0.77)	61/8,019 (0.76)	1.01 (0.61-1.68)	0.965	1.02 (0.66-1.59)	0.919
Control	2,580/2,600 (99.23)	7,958/8,019 (99.24)	1		1	
CCI scores ≥ 2 (n = 8,526)						
Crohn's disease	18/1,872 (0.96)	60/6,654 (0.9)	1.07 (0.63-1.81)	0.807	1.12 (0.71-1.74)	0.629
Control	1,854/1,872 (99.04)	6,594/6,654 (99.1)	1		1	

Abbreviations: CCI, Charlson Comorbidity Index.

* Significance at P < 0.05

† Adjusted for age, sex, income, region of residence, and CCI scores.

Supplementary Table S3. Crude and overlap propensity score weighted odd ratios of Ulcerative colitis for colorectal cancer

Characteristics	N of Colorectal cancer	N of Control	Odd ratios for Colorectal cancer (95% confidence interval)			
	(exposure/total, %)	(exposure/total, %)	Crude†	*p*	Overlap weighted model †	*p*
Total participants (n = 49,600)						
Ulcerative colitis	117/9,920 (1.18)	306/39,680 (0.77)	1.54 (1.24-1.90)	<0.001*	1.52 (1.27-1.83)	<0.001*
Control	9,803/9,920 (98.82)	39,374/39,680 (99.23)	1		1	
Age < 65 years old (n = 24,265)						
Ulcerative colitis	54/4,853 (1.11)	120/19,412 (0.62)	1.81 (1.31-2.50)	<0.001*	1.83 (1.38-2.43)	<0.001*
Control	4,799/4,853 (98.89)	19,292/19,412 (99.38)	1		1	
Age ≥ 65 years old (n = 25,335)						
Ulcerative colitis	63/5,067 (1.24)	186/20,268 (0.92)	1.36 (1.02-1.81)	0.036*	1.35 (1.06-1.72)	0.014*
Control	5,004/5,067 (98.76)	20,082/20,268 (99.08)	1		1	
Male (n = 29,665)						
Ulcerative colitis	72/5,933 (1.21)	184/23,732 (0.78)	1.57 (1.20-2.07)	0.001*	1.56 (1.24-1.98)	<0.001*
Control	5,861/5,933 (98.79)	23,548/23,732 (99.22)	1		1	
Female (n = 19,935)						
Ulcerative colitis	45/3,987 (1.13)	122/15,948 (0.76)	1.48 (1.05-2.09)	0.025*	1.47 (1.10-1.96)	0.01*
Control	3,942/3,987 (98.87)	15,826/15,948 (99.24)	1		1	
Low income group (n = 24,025)						
Ulcerative colitis	50/4,805 (1.04)	132/19,220 (0.69)	1.52 (1.10-2.11)	0.012*	1.51 (1.14-1.99)	0.004*
Control	4,755/4,805 (98.96)	19,088/19,220 (99.31)	1		1	
High income group (n = 25,575)						
Ulcerative colitis	67/5,115 (1.31)	174/20,460 (0.85)	1.55 (1.17-2.06)	0.002*	1.54 (1.21-1.96)	<0.001*
Control	5,048/5,115 (98.69)	20,286/20,460 (99.15)	1		1	
Urban resident (n = 22,235)						
Ulcerative colitis	56/4,447 (1.26)	138/17,788 (0.78)	1.63 (1.19-2.23)	0.002*	1.61 (1.23-2.11)	<0.001*
Control	4,391/4,447 (98.74)	17,650/17,788 (99.22)	1		1	
Rural resident (n = 27,365)						
Ulcerative colitis	61/5,473 (1.11)	168/21,892 (0.77)	1.46 (1.09-1.96)	0.012*	1.45 (1.13-1.86)	0.003*
Control	5,412/5,473 (98.89)	21,724/21,892 (99.23)	1		1	
CCI scores = 0 (n = 30,455)						
Ulcerative colitis	66/5,448 (1.21)	175/25,007 (0.7)	1.74 (1.31-2.31)	<0.001*	1.75 (1.38-2.22)	<0.001*
Control	5,382/5,448 (98.79)	24,832/25,007 (99.3)	1		1	
CCI scores = 1 (n = 10,619)						
Ulcerative colitis	30/2,600 (1.15)	67/8,019 (0.84)	1.39 (0.90-2.14)	0.14	1.45 (0.97-2.15)	0.067
Control	2,570/2,600 (98.85)	7,952/8,019 (99.16)	1		1	
CCI scores ≥ 2 (n = 8,526)						
Ulcerative colitis	21/1,872 (1.12)	64/6,654 (0.96)	1.17 (0.71-1.92)	0.539	1.18 (0.78-1.80)	0.43
Control	1,851/1,872 (98.88)	6,590/6,654 (99.04)	1		1	

Abbreviations: CCI, Charlson Comorbidity Index.

* Significance at $p < 0.05$

† Adjusted for age, sex, income, region of residence, and CCI scores.
